# SLC14A1 (UT-B) gene rearrangement in urothelial carcinoma of the bladder: a case report

**DOI:** 10.1186/s13000-020-01009-8

**Published:** 2020-07-23

**Authors:** Zhongying Guo, Xiaobing Niu, Guangbo Fu, Baoxue Yang, Guangping Chen, Su’an Sun

**Affiliations:** 1 Department of Pathology, Huai’an First People’s Hospital, Nanjing Medical University, Huai’an, 223300 China; 2 Department of Urology, Huai’an First People’s Hospital, Nanjing Medical University, Huai’an, 223300 China; 3 Department of Pharmacology, School of Basic Medical Sciences, Peking University, Beijing, 100191 China; 4 Department of Physiology, Emory University School of Medicine, Atlanta, GA 30322 USA

**Keywords:** Bladder, Urothelial cancer, Fluorescence in situ hybridization, Gene rearrangement

## Abstract

**Background:**

Bladder cancer (BC) is a common and deadly disease. Over the past decade, a number of genetic alterations have been reported in BC. Bladder urothelium expresses abundant urea transporter UT-B encoded by *Slc14a1* gene at 18q12.3 locus, which plays an important role in preventing high concentrated urea-caused cell injury. Early genome-wide association studies (GWAS) showed that UT-B gene mutations are genetically linked to the urothelial bladder carcinoma (UBC). In this study, we examined whether Slc*14a1* gene has been changed in UBC, which has never been reported.

**Case presentation:**

A 59-year-old male was admitted to a hospital with the complaint of gross hematuria for 6 days. Ultrasonography revealed a size of 2.8 × 1.7 cm mass lesion located on the rear wall and dome of the bladder. In cystoscopic examination, papillary tumoral lesions 3.0-cm in total diameter were seen on the left wall of the bladder and 2 cm to the left ureteric orifice. Transurethral resection of bladder tumor (TURBT) was performed. Histology showed high-grade non-muscle invasive UBC. Immunostaining was negative for Syn, CK7, CK20, Villin, and positive for HER2, BRCA1, GATA3. Using a fluorescence in situ hybridization (FISH), *Slc14a1* gene rearrangement was identified by a pair of break-apart DNA probes.

**Conclusions:**

We for the first time report a patient diagnosed with urothelial carcinoma accompanied with split *Slc14a1* gene abnormality, a crucial gene in bladder.

## Introduction

Urothelial bladder carcinoma (UBC) is the sixth most common cancer and occurs more often in men than in women. In 2004, the World Health Organization divided bladder tumors into muscle-invasive urothelial carcinoma and non-muscle invasive urothelial neoplasia [1]. More than 70% of UBC patients are non-muscle invasive (superficial) UBC at initial diagnosis, whereas 30% are in muscle-invasive stage [1]. An important clinical feature of UBC is an unusually high propensity for recurring than in any other solid cancer. More than 70% of patients with UBC will have a recurrence during the first 2 years after diagnosis [2, 3]. The recurrences are often accompanied by grade and/or stage progression with a very poor prognosis [4, 5].

In human, the urinary bladder is a special organ that is in constant contact with a high concentration of urea, 20–100 times higher than in blood. Urea is the major end-product of nitrogen metabolism and is excreted by the kidneys. Urea is a small molecule (~ 60 Da) and water solubility, however urea permeability across lipid bilayers is very low. Movement of urea across the cell membrane is mediated by urea transporter (UT) proteins [6–9]. In mammals, there are two subfamilies of UT, UT-A and UT-B, encoded by the *Slc14a2* and *Slc14a1* genes, respectively. UT-A urea transporters are mainly expressed in renal medulla and contribute to the kidney’s ability to concentrate urine. UT-B has broadly tissue expression including bladder. Bladder urothelium only expresses UT-B [10–12]. This was confirmed by an RNA-sequencing analysis showing high expression of *Slc14a1* (UT-B) and the absence of *Slc14a2* (UT-A) in bladder tissues [13].

Evidences have shown that an excess of intracellular urea accumulation damages cells and cell functions (DNA damage, abnormal cell phenotype transformation, genetic pathway activation, etc.) [11, 14–16]. Since the bladder stores urine, its urothelium is inevitably and constantly exposed to a higher concentration of urea than any other tissues. Therefore, the urothelial UT-B stands in a key position to prevent urea-induced insults by lowering intracellular urea levels. To support it, bladder urothelium expresses the most abundant urea transporter UT-B in vivo [10]. Dysfunction of UT-B will cause intracellular urea accumulation and profoundly affect cells. The impaired urothelial cells are then vulnerable to be attacked by urea and/or by carcinogen in urine, a potential important mechanism of tumorigenesis in UBC [17].

A previously study from our group revealed absence or a lower level of UT-B expression in UBC both at mRNA and protein levels. In some patients, a mutant UT-B with a 24-nt in-frame deletion (del24) in exon 4 was identified [17]. Since UT-B was undetectable in some UBC patients [17], we then asked whether this could be due to the UT-B (*Slc14a1*) gene rearrangement. We designed a pair of *Slc14a1* gene break-apart DNA probes and performed fluorescence in situ hybridization (FISH). We found UT-B gene rearrangement occurred in one UBC patient from 14 cases. This is the first report of genomic abnormalities of split SLC14A1 gene identified in UBC.

## Case presentation

A 59-year-old male was admitted to a hospital with the complaint of gross hematuria for 6 days. There was no abdominal or flank pain and other abnormal finding. No lymphadenopathy was identified. No abnormal laboratory findings were noted. The patient did not have family history of UBC. Ultrasonography revealed a size of 2.8 × 1.7 cm mass lesion located on the rear wall and dome of the bladder. Bladder tumor was diagnosed. In cystoscopic examination, papillary tumoral lesions 3.0-cm in total diameter were seen on the left wall of the bladder and 2 cm to the left ureteric orifice. Transurethral resection of bladder tumor (TURBT) was performed.

This study was conducted under the approval by the Clinical Research Ethics Committee of Huai’an Hospital, Nanjing Medical University. A signed informed consent was obtained from the patient for the use of tissue samples for this study.

### Pathologic findings

**Fig. 1:**
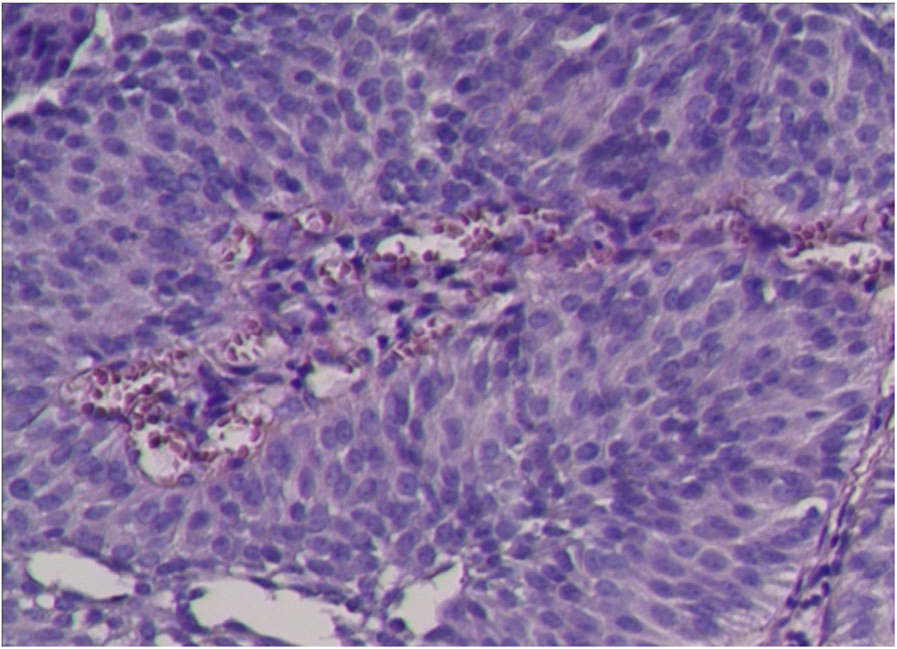
Pathological examination. A 59-year-old male complaint of gross hematuria for 6 days. Transurethral resection of bladder tumor (TURBT) was performed. Histology shows typical morphology of bladder urothelial cancer

The resected tissues were sent to the pathologic lab and fixed in 10% formalin. Paraffin tissue blocks were made as standard technique, and 3 μm sections were prepared for pathological diagnosis, immunohistochemistry (IHC), and FISH test. Hematoxylin and eosin (HE) stained slides were reviewed independently by two senior pathologists. Pathological examination showed high-grade non-muscle invasive papillary urothelial carcinoma (Fig. 1).

### Immunohistochemical findings

Immunohistochemistry analysis showed HER2 (3+), Syn (−), BRCA1(2+), GATA3 (3+), Ki-67 (30%+), CK7 (−), CK20 (−), Villin (−), p53 (±).

### Molecular findings

**Fig. 2:**
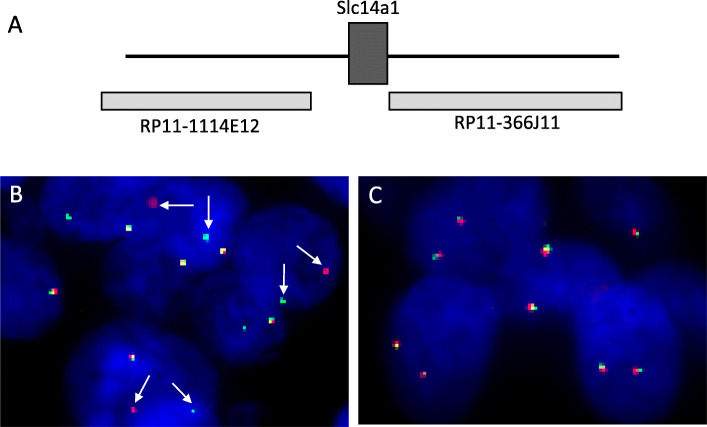
FISH analysis using “break-apart” Slc14a1 gene probes. **a**. Illustration of FISH probe design and probe location. **b**. UT-B gene split was identified showing separated red dots and green dots (arrows) in cancer cells. **c**. Control. A representative image of UBC cell without UT-B gene split from a different patient. Normal signals are two close red and green colors with partial overlapped yellow color

FISH was performed using *Slc14a1* break-apart rearrangement dual color probe in this experiment. The probe consists of two combinations, a ~ 200 kb of DNA (RP11-1114E12) at the centromeric side of *Slc14a1* gene being labelled by the tetramethyl rhodamine with a red fluorescence signal, a ~ 190 kb of DNA (RP11-366 J11) located in the telomeric side being marked by green fluorescence (Fig. 2a). RP11 bacterial artificial chromosome (BAC) clones with human genomic DNA were purchased from UCSF Benioff Children’s Hospital Oakland. Plasmid DNAs were prepared by NucleoBond Xtra BAC large construct kit (Takara, 740,436.25). DNA probes were fluorescent labelled using Nick translation DNA labeling system (Enzo-GEN111).

Three micrometer thick sections were de-paraffinized in xylene, dehydrated with ethanol, pre-treated in 90 °C water for 30 min, and then incubated with 1 mg/ml pepsin (Amresco) solution at 42 °C for 15 min. The break-apart *Slc14a1* probe set including human Cot-1 DNA was applied to individual slides. Human Cot-1 DNA (Fisher, 15,279,011) was used to block non-specific sequences. After denatured at 86 °C for 8 min, the sections were incubated for 16 h at 38 °C using Automated ThermoBrite FISH System (Leica Biosystems). Post-hybridization washes were carried out with 2 × SSC for 3 min at room temperature followed by 2X SSC/0.1% NP-40 solution at 72 °C for 5 min and gradient dehydration in 70, 85 and 100% ethanol. The sections were air-dried, protected from light and mounted with Fluorescence Mounting Medium containing DAPI (Sigma F6057). The fluorescence was viewed under Olympus FV1000 Upright fluorescence microscope. The fluorescence signals were evaluated in 50 nuclei for each tumor core under a 100x immersion objective. According to Noda’s diagnostic criteria [18] for MAML2 gene split in mucoepidermoid carcinoma, we considered the positive *Slc14a1* rearrangement if > 10 split signals of 50 nuclei were found.

The hybridization signals of *Slc14a1* break-apart probe showed two very close red and green colors with partially overlapped yellow colors indicating normal unbroken *Slc14a1* gene. Figure 2c showed a representative image of FISH results without UT-B gene split. When the *Slc14a1* gene break apart occurred, the signals are changed from merged yellow to single red and green as positive of UT-B gene split. From total 14 cases of UBC patients, we found that one patient (case report in this study) demonstrated break-apart signal patterns of *Slc14a1* gene (Fig. 2b).

## Discussion

UBC is a complex and heterogeneous disease caused by both genetic and environmental factors. The “urogenous contact hypothesis” proposed by Braver [19] in 1987 is still prevalent in the etiology of UBC. However, one important factor that we may have ignored in bladder carcinogenesis is urea. The urea concentration in urine is 20–100 times higher than that in blood in humans [20, 21]. Interestingly, the risk of UBC for people with spinal cord injuries is 16–28 times higher than that for the general population [22, 23], underscoring the important role of endogenous factors, including urea, in the bladder tumorigenesis.

Considering that the bladder urothelium is bathed in fluid with a high urea concentration and the toxicity of intracellular urea accumulation, the urea transporter is extremely important for bladder urothelium by lowering intracellular urea levels. Experimental studies have shown that deletion of UT-B gene causes urea accumulation in urothelium ~ 9 times higher in UT-B knockout mice [11, 24]. This is accompanied by increased cell DNA damage, apoptosis and diminished arginine metabolism [11, 24]. Therefore, loss of UT-B protection will render urothelial cells at risk under constantly high urea insult, then either directly activates genetic pathways that induce UBC or synergistically increases UBC risk by facilitating other carcinogen-induced mechanisms of UBC. In 2011, genome-wide association studies (GWAS) from two independent groups [25, 26] reported a number of gene variants of nucleotide polymorphisms within SLC14A1 (UT-B gene) on chromosome 18q12.3, are associated with risk of developing UBC in humans, suggesting the possible tumor suppressor role of UT-B in UBC.

Genomic alterations are quite common in UBC [1]. Previous gene analysis revealed three types of UT-B gene abnormality in UBC [17, 1) no or under-detectable mRNA expression; (2) truncated short form of UT-B; (3) UT-B with a 24-nt in-frame deletion (del24) in exon 4 (UT-BΔ24). In this study, we attempted to figure out whether in some UBC patients the absence of UT-B mRNA expression [17] measured by RT-PCR is due to *Slc14a1* gene rearrangement. Using a set of *Slc14a1* break apart DNA probes, we performed FISH test by using FFPE tissue sections from 14 UBC patients. A patient of a 59-year-old male diagnosed with superficial, non-muscle invasive UBC clearly showed split *Slc14a1* gene signals (Fig. 2b). Gene split has been demonstrated in several cancers [27–29]. This is the first report demonstrating a case of UBC with *Slc14a1* gene rearrangement.

## Conclusions

Although the *Slc14a1* as a new urinary UBC susceptibility gene was appreciated [25, 26], the role and the molecular mechanism of the UT-B in bladder oncogenesis have not been well explored to date. The discovery of *Slc14a1* gene rearrangement in this study is interesting and may have clinical significance. Further investigation is needed to substantiate this finding and determine whether the *Slc14a1* gene rearrangement is associated with tumor cell invasion, metastatic spread, aggressiveness, tumor progression, recurrence and clinical outcomes.

## Data Availability

The datasets used and/or analyzed during the current study available from the corresponding author on reasonable request.

## Abbreviations

BC: Bladder cancer
UBC: Urothelial bladder carcinoma
UT: Urea transporter
GWAS: Genome-wide association studies
FISH: Fluorescence in situ hybridization
IHC: Immunohistochemistry
